# Altered proteolytic events in experimental autoimmune encephalomyelitis discovered by iTRAQ shotgun proteomics analysis of spinal cord

**DOI:** 10.1186/1477-5956-7-25

**Published:** 2009-07-16

**Authors:** Mohit Raja Jain, Shengjie Bian, Tong Liu, Jun Hu, Stella Elkabes, Hong Li

**Affiliations:** 1 Center for Advanced Proteomics Research and Department of Biochemistry and Molecular Biology, UMDNJ-New Jersey Medical School Cancer Research Center, Newark, NJ 07103, USA; 2 Department of Neurology and Neuroscience, UMDNJ-New Jersey Medical School, Newark, NJ 07103, USA

## Abstract

**Background:**

Abnormal activation of protease activities during experimental autoimmune encephalomyelitis (EAE) in rats, a rodent model of multiple sclerosis, have been implicated in either the direct destruction of myelin components or the intracellular signal transduction pathways that lead to lymphocyte infiltration, oligodendrocyte destruction, neuronal dysfunctions and axonal degeneration. The identification of changes in regulated proteolytic events during EAE is crucial for uncovering activated proteases that may underline the pathological features such as inflammation and demyelination. We searched for either non-tryptic or semi-tryptic peptides from a previous shotgun proteomics study using isobaric tags for relative and absolute quantification (iTRAQ) to compare the proteomes of normal and EAE rat lumbar spinal cords.

**Results:**

We discovered that several proteins, such as α_1_-macroglobulin, a protease inhibitor, α_1_B-glycoprotein, β_2_-microglobulin, neurofilament light polypeptide and sulfated glycoprotein 1 had non-tryptic peptide iTRAQ ratios that were substantially different from the overall protein iTRAQ ratios, suggesting that such peptides may be markers for the proteolytic products generated by the protease(s) altered during EAE. Indeed, subsequent Western blotting confirmed the dysregulation of specific protein cleavages in EAE tissues. Additional proteolytic changes in α_2_-macroglobulin, another protease inhibitor similar to α_1_-macroglobulin was also observed.

**Conclusion:**

The results from this study revealed changes among both neuronal protein processing and endogenous proteolysis modulators in EAE animals. This information may provide a rationale for protease inhibitor-based therapeutic interventions for multiple sclerosis.

## Background

Proteases and peptidases are important regulators that govern many cellular functions [1]. Some protease activities are manifested globally, e.g. during protein turnover in lysosomes and proteasomes. Other proteases are activated only within particular defined contexts, serving specific signal transduction and other regulatory functions. Well-known examples include the caspase cascade during apoptosis, the coagulation cascade during clot formation and the classical and alternative complement innate immune systems for pathogen clearance. More recently, regulated proteolysis events have been implicated in numerous disease-related processes, including calpain activation following excitotoxicity in neuronal cells [2], matrix metalloprotease (MMP) modulation during cancer metastasis and multiple sclerosis [3], and the contribution of rennin and angiotensin converting enzyme to regulate blood pressure and cardiovascular function [4]. Understanding how protease activities are regulated is important both for discerning basic biological mechanisms and for developing therapies that can regulate pathological protease activities.

Quantitative proteomics techniques have been developed to uncover regulated proteolysis events (i.e. the identification of the cleavage sites within targeted proteins) and possibly identify the responsible protease(s). These methods can be broadly divided into following categories: 1) quantification of changes among selective proteases; 2) quantification of known protease substrates; 3) selective quantification of protein N-termini. For example, Cravatt *et al*. pioneered an activity-based proteomics technique to use selective inhibitors for affinity enrichment and quantification of proteases [5]. Overall *et al*. developed several strategies to quantify changes in known or putative protease substrates in cells and tissues expressing different levels of MMPs with great success [6,7]. In particular, their work has utilized the iTRAQ technique for the identification of protease substrates and determination of proteolytic sites [8]. In line with the general theme that sub-proteome analyses are usually more sensitive than global proteomic studies for specific biological objectives. Van Damme *et al*. refined a technique called combined fractional diagonal chromatography to separate N-terminal peptides from their internal tryptic counterparts for more sensitive degradomic analysis [9]. Recently, Wells group demonstrated an elegant technique for global identification of proteolytic cleavage sites within proteins in apoptotic cells by specific labeling of protein N-termini [10]. After, selectively biotinylating the α-amines of N-termini of peptides (including neo N-termini produced during apoptosis) with subtiligase, derivatized tryptic peptides were enriched on an avidin media, and the sites of proteolytic cleavage were identified by LC-MS/MS. All of these specialized methods and experimental designs have been proven effective for revealing different aspects of the degradome. Given the complexities involved in biological systems, however, it may not always be possible to predict, whether altered proteolytic events are important for a study in which proteomics analysis is needed. Because of this, there are many quantitative data that have already been obtained for expression proteomics studies could contain valuable information on altered proteolytic events that are critical for the underlying biological scenarios. In this report, we demonstrate a simple method to uncover regulated proteolytic events from a quantitative shotgun proteomics dataset obtained using the iTRAQ technique [11]. The validity of our current approach is based on the assumptions that: 1) regulated proteolytic activities are typically non-tryptic, so the resulting peptides would be either semi- or non-tryptic and 2) the iTRAQ expression ratios for these peptides are different from those of the tryptic peptides derived from the same protein. Obviously, these assumptions do not apply to the targets of those proteases whose substrate specificities overlap with trypsin.

The model systems used in the present study were the spinal cords isolated from rats suffering from EAE, a well-characterized animal model of multiple sclerosis [4,11]. This human neurodegenerative autoimmune disease is characterized by inflammation, demyelination in the central nervous system (CNS), axonal damage and neuronal loss leading to neurological deficits such as paresis and paralysis. Since the mechanisms underlying the damages to CNS cells in this disease remain elusive, we have previously utilized the iTRAQ technology to define differentially expressed and post-translationally modified proteins during acute EAE in the Lewis rat lumbar spinal cord, the region most affected in this model [11,12]. Elevated levels of proteases such as MMPs have previously been suggested to play a role in mediating EAE pathology [4], and functional inhibition of selective MMP activities has been shown to alleviate EAE symptoms [4]. Interestingly, inhibition of MMP-2 and MMP-12 made some animals more susceptible to EAE [13], suggesting that the proteolytic activity in EAE is rather complex and providing a rationale for further analysis of the EAE degradome. In this study, we were able to identify changes in regulated proteolysis events in EAE via searching for non-tryptic and semi-tryptic peptides. By comparing the iTRAQ expression ratios of the semi and non-tryptic fragments with their putative protein expression ratios averaged from the iTRAQ ratio of all tryptic peptide, we were able to uncover changes in selective CNS proteins and endogenous protease inhibitors. These results were in accord with known protease dysregulations reported in tissues of both EAE animals and multiple sclerosis patients. This additional information clearly complements differential expression data commonly sought after by those performing shotgun proteomics studies and it can be obtained with relatively little additional effort and cost, thus improving proteomic research productivity.

## Results

### Identification of Differentially Cleaved Protein Products

**Figure 1:**
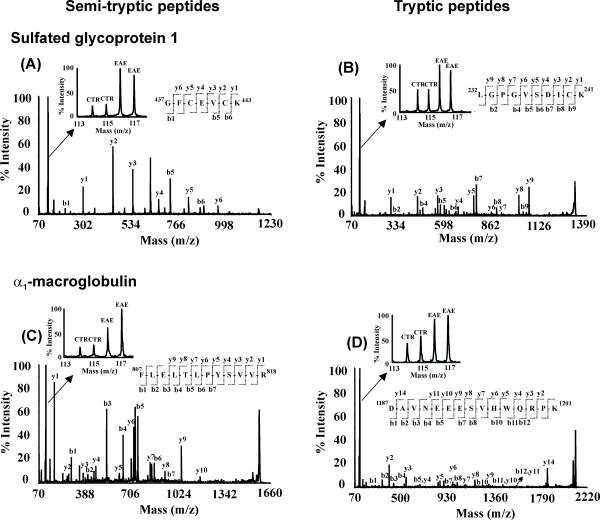
**MS/MS spectra of representative semi-tryptic and tryptic peptides**. iTRAQ reporter ion region and peptide sequencing region of the MS/MS spectrum for semitryptic peptides derived from (A) sulfated glycoprotein 1 (437–443) and (C) α_1_-macroglobulin (807–818). Representative tryptic peptides changes are shown for (B) sulfated glycoprotein 1(232–241) and (D) α_1_-macroglobulin (1187–1201). Peptide sequences were deduced from the MS/MS spectra based on the observation of continuous series of either N-terminal (b-series) or C-terminal (y-series) ions. The peak areas of iTRAQ quantification (shown in insets of A, B, C, D) ions, m/z 114–117 were used to measure the relative abundance of individual peptides.

In our previous study, we reported the identification of 41 differentially expressed proteins [11] and changes in citrullination, methylation and phosphorylation of various proteins [12] in the spinal cord of EAE animals. To our surprise, we also discovered the increased levels of a distinct proteolytic fragment (~50 kDa) of moesin in EAE spinal cords, following Western blotting validation analysis of iTRAQ quantitation results [11], suggesting that there may be other proteolytic products present in EAE animal tissues. In the current study, we examined proteins that were differentially cleaved in EAE animals. We identified 197 semi-tryptic or non-tryptic peptides belonging to 104 proteins (see Additional file 1). Among these, we found seven proteolytic products whose levels were significantly altered in EAE compared to control animals, although the corresponding total protein levels were not changed to the same degrees (Table 1, see Additional files 2 and 3). For example, a semi-tryptic peptide (^437^GFCEVCK^443^) from sulfated glycoprotein 1, produced from N-terminal non tryptic cleavage between G436 and G437, was found to be dramatically increased in EAE tissues and had an iTRAQ ratio of 4.5 (Fig. 1A). However, its total protein level deduced from all the tryptic peptides (see Additional file 2) observed for this protein was altered to a lesser extent (EAE/control ratio of 1.9, Table 1), as demonstrated by the iTRAQ reporter ion regions for a representative tryptic peptide ^68^TVVTEAGNLLK^78 ^for this protein (Table 1 and Fig. 1B). Similarly, a semi-tryptic peptide ^807^FLELTLPYSVVR^818 ^from α_1_-macroglobulin (α_1_M) (Fig. 1C) was found to be significantly elevated with an iTRAQ ratio of 5.9 in the EAE tissues; however, the total α_1_M protein level was increased with an iTRAQ ratio of 2.8 (Table 1 and Fig. 1D). Likewise, another semi-tryptic peptide ^84^ILAHTEFTPTETDVYACR^101 ^from β_2_-microgloublin was found to be increased with an iTRAQ ratio of 3.1 in the EAE tissue as compared to an iTRAQ ratio of 2.7 in total β_2_-microgloublin protein level (Table 1). Interestingly, the semi-tryptic peptides from both neurofilament light polypeptide (^443^YTSHVQEEQSEVEETIEATK^463^) and phosphoglucomutase-1 (^184^TVEIVDSVEAYATMLR^200^) were higher (iTRAQ ratios of 2.2 and 1.6 respectively) in EAE tissues. However, no significant changes were observed in their protein levels (iTRAQ ratios of 0.9 and 1.1 respectively, Table 1) in EAE tissues.

**Table 1:** Altered non-tryptic cleaved peptides in EAE rat spinal cord

**Protein**	**Swiss-Prot Accession Number**	**Peptide**^a^	**Observed Mass (m/z)**	**Error (ppm)**	**Peptide Ratio^b ^± SD**	**p-Value**	**Protein Ratio (N)**^c^	**Sequence Coverage (%)**
**α**_1_B-glycoprotein	Q9EPH1|A1BG	GPGNA-^21^LWLDSGSEPELR^32^-AEPQS	1545.743	38	4.3 ± 1.2	0.02	3.3 (5)	6

α_1_-Macroglobulin	Q63041|A1M	VFQPF-^807^FLELTLPYSVVR^818^-GEAFI	1580.917	0	5.9 ± 1.6	0.03	2.8 (19)	13

β_2_-Microglobulin	P07151|B2MG	DWSFY-^83^ILAHTEFTPTETDVYACR^101^-VKHVT	2257.067	4	3.1 ± 0.8	0.02	2.7 (2)	26

Neurofilament light polypeptide	P19527|NFL	AFPAY-^443^YTSHVQEEQSEVEETIEATK^463^-AEEAK	2625.292	6	2.2 ± 0.6	0.05	0.9 (75)	56

Phospho glucomutase-1	P38652|PGM1	KFKPF-^184^TVEIVDSVEAYATMLR^200^-NIFDF	1941.006	-3	1.6 ± 0.1	0.01	1.1 (6)	17

Sulfated glycoprotein 1	P10960|SAP	QPKAN-^194^EDVCQDCMK^202^-LVTDI	1450.59	11	2.4 ± 0.2	0.00	1.9 (16)	19

Sulfated glycoprotein 1	P10960|SAP	PQKNG-^437^GFCEVCK^443^-KLVIY	1165.508	-3	4.5 ± 0.7	0.00	1.9 (16)	19

^a ^Sequences bracketed by amino acid numbers are the semi-tryptic peptides discovered experimentally. Five amino acids before and after the semi-tryptic peptides are shown to indicate the non tryptic cleavage sites.

^b ^iTRAQ Ratio: EAE/Control

^c ^Total number of peptides identified for respective protein (see Additional file 2)

### Corroboration of iTRAQ analysis by Western Blot

**Figure 2:**
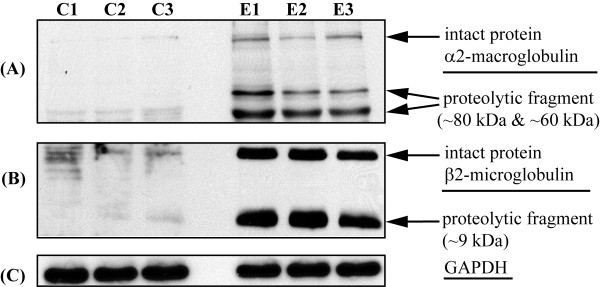
**Western blot validation of select protein cleavages**. (A) α_2_-macroglobulin, (B) β_2_-microglobulin from both control (C1, C2, C3) and EAE (E1, E2, E3) animals. (C) GAPDH was used to determine the equal loading of proteins for all the samples.

In this study, we observed that several proteins previously implicated in EAE etiology were differentially processed. A highly specific antibody for rat α_1_M is not commercially available for validation. However, rat α-macroglobulins 1 and 2 (α_2_M) are similar in their amino acid sequence (56% of sequence homology, see Additional file 4) and have been suggested to have comparable three-dimensional structures and function [14]. We tested whether α_2_M may also be similarly processed as with α_1_M in EAE by analyzing the presence of both breakdown products using an antibody against α_2_M (Fig. 2A). Interestingly, the levels of both intact α_2_M (~165 kDa) and a proteolytic product (~80 kDa) were elevated in the EAE tissues than the controls (Fig. 2A). Another α_2_M proteolytic product (~60 kDa) levels were also found to be increased in the EAE tissues (Fig. 2A). Using a longer exposure of the Western blotting film, more proteolytic fragments can be observed in the EAE tissues (see Additional file 5). We further probed for the presence of proteolytic products from other proteins for which the specific antibodies are commercially available. In the case of β_2_-microglobulin, which was increased in the EAE tissues (iTRAQ ratio of 2.7, Table 1), we found that the levels of both the intact protein as well as its proteolytic fragment were dramatically increased in the EAE tissues by Western blotting (Fig. 2B), corroborating the observations made from the iTRAQ experiment. We also probed for the presence of proteolytic products of sulfated glycoprotein 1. The levels of the intact protein (~62 kDa) were found to be increased in the EAE tissues (see Additional file 5), corroborating iTRAQ results (Table 1). However, we could not find the presence of its proteolytic products, which may be indicative of either rapid degradation of the proteolytic products or changes in the epitopes for the antibody used. The neurofilament light polypeptide was found unchanged in the EAE tissues by iTRAQ analysis (Table 1). Using an anti-neurofilament light polypeptide antibody, we confirmed that intact neurofilament light polypeptide (~68 kDa) was not changed in the EAE tissues (see Additional file 5); however, by comparison, a putative proteolytic product (~11 kDa) was increased by ~10% in the EAE tissues (see Additional file 5).

## Discussion

There are many techniques for the analysis of the degradome. Although the methods employed here do not incorporate enrichment techniques, we were able to identify cleaved proteins. Although we assumed only non-tryptic fragments as markers for cleaved proteins in the spinal cord of rats affected by EAE, there are proteases – especially in the serine protease family – whose substrate cleavage specificities may overlap with trypsin. In principle, such cleavages may also be identified by iTRAQ analysis, but it is difficult to distinguish endogenous cleavages from tryptic cleavages of extracted proteins. Under such circumstances, the techniques developed by McDonald *et al *[15] and Ji *et al *[16] to first block the protein N-termini prior to tryptic digestion and enrichment of the neo-N-termini should be used to discover the proteolytically modulated proteins. Overall, we have observed nearly 200 non or semi-tryptic events in over 100 proteins. It is likely that significantly more such events could be detected with focused sub-proteomic analysis of the degradomes, using the approaches elegantly described by Wells [10], Van Damme [9] and others. However, since most expression proteomics experiments have been conducted without the enrichment of the protein N-termini, it is important to be able to extract differential proteolytic signals from such studies in addition to protein expression information, considering the significant amount of resources devoted for such experiments. We have demonstrated here that it is possible to obtain such information with bioinformatic data reprocessing. The existence of differential proteolysis in the expression proteomic datasets may provide a rationale for subsequently more focused degradomic studies.

EAE is an established animal model for studying cellular pathways leading to demyelination, axonal damage, lymphocyte infiltration of the CNS, and other processes that occur in CNS of multiple sclerosis patients [17-19]. Our previous iTRAQ analysis of the spinal cord in acute EAE revealed 41 differentially expressed proteins, including complement C3, α_2_M, ceruloplasmin, and other acute phase proteins commonly associated with systematic inflammation [11]. During different stages of multiple sclerosis and EAE, aberrant proteolytic processes have been reported to be involved in axonal damage, oligodendrocyte apoptosis, demyelination, pro-inflammatory cytokine activation, epitope spreading, T cell and macrophage activation, and damage to the blood brain barrier [4]. Although some of these proteolytic events involve tissue triage and broader damage, under most circumstances, the implicated proteolytic events involve limited cleavages at specific peptide bonds [4], suggesting the existence of regulated proteolytic events. The dysregulation of proteolysis in EAE pathology may be dependent on protease levels, localization, activation and endogenous inhibitor concentrations [4]. For example, MMPs including MMP-9, 2, 14, 3, 7, 12, 8 have been reported to be elevated at different stages of multiple sclerosis [4,13], and they are important promoters of immune cell extravasation into the CNS *via *the opening of the blood brain barrier due to their unique ability to facilitate fibronolysis [20]. In addition to MMPs and their inhibitors, serine proteases, such as tissue plasminogen activator (tPA), urokinase plasminogen activator, thrombin, elastase, tissue kallikreins and their inhibitors have also been reported to be activated in various multiple sclerosis patients and animal models [4].

Among the proteins that were found to have increased proteolytic cleavage products is α_1_M, also known as pregnancy zone protein. It is a widely expressed plasma glycoprotein protease inhibitor that belongs to the α_2_M family [21]. These protease inhibitors are synthesized in the liver and form dimeric and tetrameric complexes *in vivo*. α_1_M has been shown to be cleaved by both MMP-2 and MMP-9, and it is capable of inhibiting mast cell tryptase, tPA, chymotrypsin and snake venom metalloprotease [22,23]. These protease inhibitors function by forming thiol esters with protease side chains following their cleavage by the proteases with a "bait region" and inhibiting protease activity towards other high molecular weight substrates [24]. α_1_M has been shown to selectively inhibit T-cell activation and IL-2 secretion [25] and also binds to both TGF-β1 and TGF-β2 [26], inhibiting their association with their cell surface receptors, which may be protective for EAE animals [25]. Given the functional significance of α_1_M, the observation of the increase of its cleavage products in EAE suggests that it may play a protective role by dampening the effect of harmful proteases. Similarly, α_2_M has been shown by Western blot analysis to be cleaved (increase in an 80 kDa fragment) in EAE spinal cords. Our previous studies have shown a significant increase (iTRAQ ratio of 2.3) in α_2_M protein levels [11]. α_2_M can be produced by activated macrophages, has been shown to attenuate EAE symptoms when administered exogenously, and may exert its beneficial effects by either neutralizing proteinases involved in tissue damage or directly interfering with antigen recognition due to its ability to bind myelin basic protein [24]. EAE is an inflammatory autoimmune condition in affected animals. It is interesting to see that several immune system proteins were differentially cleaved in EAE. β_2_-microglobulin is part of MHC class I molecule that is important for antigen presentation [27]. Neurofilament light polypeptide has been reported to be degraded in EAE animals putatively by calpain [28] and possibly the result of increased oxidative stress during EAE [29]. Based on our proteomics study, it appears that proteins related to neuroinflammation, neuroregeneration and axonal integrity may proteolytically processed in EAE. Further studies are needed to determine the functional significance of these cleaved proteins.

## Conclusion

Proteolysis is an important means of post-translational regulation of neuronal cell function; its dysregulation may underlie the pathology of EAE and multiple sclerosis. Many of the implicated proteases are important regulator of cytokines and chemokines [4]. Changes in different protease inhibitors discovered in this study, like α_1_M and α_2_M (Table 1) and their proteolytic fragments have been reported in recent proteomics searches for clinical biomarkers. For example, a cleaved product of cystatin C, an inhibitor of cysteine proteases has been reported as a potential biomarker in the cerebrospinal fluid of multiple sclerosis patients [30], although the validity of this peptide as disease biomarker has recently been challenged by another study [31]. The results from our current study revealed changes among both neuronal protein processing and endogenous proteolysis modulators. This information may provide a rationale for further studies to develop protease inhibitor-based therapeutic interventions for demyelinating diseases and multiple sclerosis.

## Methods

### Induction of EAE

Two month-old Lewis rats were immunized with myelin basic protein (MBP) emulsified in complete Freund's adjuvant (CFA) or CFA-containing vehicle according to Nicot *et al *[32]. Rats were maintained in a standard 12 h light/dark cycle and had free access to water and food based on approved IACUC protocols. EAE clinical symptoms were scored as follows: 1, tail weakness; 2, hind limb weakness; 3, hind limb paralysis; 4, quadriplegia; and 5, moribund. For this study, lumbar spinal cords (region most affected by EAE) were harvested when EAE-induced rats exhibited hind limb paralysis (clinical score 3). The dissected lumbar spinal cords were immediately frozen on dry ice and stored at -80°C until further use.

### Protein extraction and iTRAQ labeling

For iTRAQ analysis, detailed methods have been described previously [11]. Briefly, lumbar spinal cords obtained from two CFA-treated controls and two rats affected by EAE were used. Fifteen milligrams of the spinal cord tissues were homogenized in 300 μl of a lysis buffer consisting of 25 mM triethylammonium bicarbonate, 20 mM sodium carbonate and 2 μl of protease inhibitor cocktail (Sigma, St Louis, MO, USA). The supernatant was cleared by centrifugation at 19,000 × g for 30 min, and the pH was adjusted to 8.0 with 0.1 M HEPES. The iTRAQ labeling procedures were performed according to the manufacturer's instructions (Applied Biosystems (ABI), Foster City, CA). Ninety micrograms of soluble proteins from each sample was reduced by the addition of 2 μl of the reducing agent, tris (2-carboxyethyl) phosphine hydrochloride (TCEP) and incubated at 60°C for 1 h. Reduced cysteines were then alkylated with the addition of 1 μl of 200 mM methyl methanethiosulfonate (MMTS) and incubated at room temperature for 10 min. To initiate tryptic digestion, 10 μg of trypsin (Promega Corporation, Madison, WI USA) was added to each of the four samples and incubated at 37°C overnight. The resulting peptides were labeled with the appropriate iTRAQ reagents. Samples derived from two different control spinal cords were labeled with iTRAQ tags 114 and 115, whereas samples obtained from two independent EAE spinal cords were labeled with tags 116 and 117. The labeled samples were then mixed together and fractionated via a strong cation exchange chromatography (SCX) on a BioCAD™ Perfusion Chromatography System (ABI), equipped with a polysulfoethyl A column (4.6 mm × 200 mm, 5 μm, 300 A°, Poly LC Inc., Columbia, MD, USA) and an upstream guard column (4.0 mm × 10 mm, Poly LC). The peptide mixture was separated with a gradient consisting of mobile phase A, containing 10 mM KH_2_PO_4 _and 20% acetonitrile (ACN) (pH 3.0), and mobile phase B, consisting of 600 mM KCl, 10 mM KH_2_PO_4 _and 20% ACN (pH 3.0). Labeled peptides were eluted with a 40-min linear gradient from 0 to 50% B, followed by another 10 min from 50 to 100% B. Two-minute fractions were dried via speed-vac and desalted via PepClean™ C_18 _spin columns (Pierce, Rockford, IL, USA). Desalted peptides were further fractionated on an Ultimate™ Chromatography System equipped with a Probot matrix-assisted laser desorption ionization (MALDI) spotting device (Dionex, Sunnyvale, CA, USA). Peptides were first captured onto a reversed phase trapping column (0.3 mm × 5.0 mm) and then resolved on a 0.1 mm × 150 mm capillary PepMap column (3 um, 100 A°, C_18_, Dionex), with a 70-min gradient of solvent A (5% ACN, 0.1% trifluoroacetic acid, TFA) and solvent B (95% CAN and 0.1% TFA): 0–4 min, from 5 to 8% B; at 34 min, to 18% B; at 57 min, to 35% B and at 64 min, to 95% B. The HPLC eluent was mixed in a 1:3 ratio with a MALDI matrix solution (7 mg/ml alpha-cyano-4-hydroxycinnamic acid, in 60% ACN, 5 mM of ammonium monobasic phosphate and the internal mass calibrants, 50 fmol/μl each of GFP and ACTH, 18–39) through a 30 nl mixing tee and spotted onto the MALDI plates in an 18×18 spot array format. The peptides were analyzed on a 4700 Proteomics Analyzer MALDI-TOF-TOF tandem mass spectrometer (ABI) in a data-dependent fashion using a job-wide interpretation method. MS spectra (m/z 800-3,600) were acquired in positive ion reflector mode with internal mass calibration. A maximum of the ten most intense ions (S/N > 50) per spot were selected for subsequent MS/MS analysis in 1 k eV mode. Each spectrum was averaged over 4,000 laser shots.

### Protein database search and bioinformatics

For automated peptide identification, ProteinPilot software (v. 2.0.1, Revision 67128, ABI) was used to process the tandem mass spectra to generate the peak lists for database search, using default parameters optimized by the manufacturer. For peptide identification, ProteinPilot uses both MS and MS/MS mass error tolerance based on established mass accuracy performance of the 4700 Proteomics Analyzer MALDI-TOF-TOF tandem mass spectrometer (ABI). The peak list was submitted for a "thorough" search against the rat sequences in the UniProtKB/Swiss-Prot v54.8 database (Release date Feb 05, 2008, 349,480 sequence entries) using the Paragon algorithm [33] with default parameters. The following search parameters were used: trypsin as a digesting agent, iTRAQ-labeled N-termini and lysines and MMTS-labeled cysteines were set as fixed modifications; oxidized methionines and iTRAQ-labeled tyrosines were set as variable modifications. The Paragon algorithm automatically search for semi- and non-tryptic peptides in addition to tryptic ones. The semi-tryptic peptides identified with confidence interval (CI) values ≥ 99% and MS measurement error ≤ 50 ppm were used for the degradome analysis. To reduce the probability of false identification, we chose to report only proteins containing at least two peptides with confidence values ≥ 95%. Semi-tryptic peptides were also verified by manual examination of the spectra (see Additional file 3). The false discovery rate (FDR) was estimated by researching the spectra against a target decoy database containing both forward and reverse rat protein entries [34]. The FDR for this study was estimated to be 5.0%. Relative quantification of peptides in each sample was calculated from the areas under the peaks at *m/z *114.1, 115.1, 116.1 and 117.1. The calculated peak area ratios were corrected for overlapping isotopic contributions as per manufacturer's instructions. If peptides identified are common to different isoforms of related proteins, Pro Group™ Algorithm (ABI), a component within the ProteinPilot software, was used to calculate protein ratios using only iTRAQ ratios from the peptides that were distinct to each isoform and thus provide isoform-specific quantitation. P-values were derived via the 1-tailed Student's t-test for each peptide by comparing the two EAE with the two control values using Excel (Microsoft Corporation, Redmond, WA).

### Western blotting analysis

Three independent control and EAE samples were used for Western blotting analysis. Thirty micrograms of proteins from each sample were first resolved on a 12.5% SDS-PAGE gel and then transferred to a nitrocellulose membrane (Bio-Rad, Hercules, CA, USA). The membrane was rinsed with PBS and the non specific binding sites were blocked in a solution of 5% nonfat milk in PBST (0.05% Tween 20 in PBS) for 1 h at room temperature, followed by three washes in PBST for 10 min each. The membrane was first incubated with rabbit α_2_-M antibody (ab58703, Abcam Inc. Cambridge, MA, USA) (1:1,000 dilution) overnight and then washed in PBST buffer as described above. The immunocomplexes were visualized by Western Lightning^® ^Western Blot Chemiluminescence Reagent Plus (PerkinElmer, Waltham, MA, USA), using goat anti-rabbit IgG coupled to horseradish peroxidase as the secondary antibody (170–6515, Bio-Rad, Hercules, CA, USA). The membrane was stripped and re-probed with either mouse anti-glyceraldehyde-3-phosphate dehydrogenase (10R-G109A, Fitzgerald, Concord, MA, USA) (1:10,000 dilution), or anti-neurofilament light polypeptide (sc-12980, Santa cruz biotechnology, Santa Cruz, CA, USA) (1:500 dilution), or anti-β_2_-microgloblulin (sc-69963, Santa cruz biotechnology, Santa Cruz, CA, USA) (1:500 dilution) and or anti-sulfated glycoprotein 1 (ab68466, Abcam Inc. Cambridge, MA, USA) (1:1,000 dilution).

## Abbreviations

EAE: experimental autoimmune encephalomyelitis; iTRAQ: isobaric tags for relative and absolute quantification.

## Competing interests

The authors declare that they have no competing interests.

## Authors' contributions

MRJ, SB, TL have performed the mass spectrometry, Western blot, statistical analysis and manuscript draft preparation. JH performed the bioinformatics. SE performed the EAE induction in animals, participated in discussion and manuscript preparation. HL conceived, designed the study and drafted the manuscript. All authors edited the manuscript and approved the final version.

## Supplementary Material

Additional file 1 **List of identified peptides with semi-tryptic cleavages**. ProteinPilot software (ABI) was used to process the tandem mass spectra and generate peak lists for the database search. Peptides identified with confidence interval values ≥ 99% are reported here. Click here for file

Additional file 2 **List of all the peptides identified for the proteins listed in Table **1. ProteinPilot software (ABI) was used to process the tandem mass spectra and generate peak lists for the database search. Peptides identified with the confidence interval values ≥ 95% are reported here and were used for calculation of the protein expression ratios. Click here for file

Additional file 3 **MS/MS spectra of the semi-tryptic peptides listed in Table **1. All MS/MS spectra were acquired on a 4700 MALDI TOF/TOF tandem MS instrument (ABI). The spectra were matched to proteins by ProteinPilot software (ABI). Click here for file

Additional file 4 **Amino acid sequence alignment of the α_1 _and α_2 _macroglobulins**. The alignment was performed using ClustalW. Rat α_1 _and α_2 _macroglobulins showing ~56% of sequence homology. Click here for file

Additional file 5 **Western blotting analyses**. (A) Longer exposure image of α_2_-macroglobulin as shown in Fig. 2A. Low levels of the α_2_-macroglobulin fragments were present in the control spinal cords. More fragments can be seen in EAE samples (B) Sulfated glycoprotein 1 from both control (C1, C2, C3) and EAE (E1, E2, E3) animals. (C) Neurofilament light polypeptide Western blotting and (D) densitometry quantification of neurofilament light protein fragments signals. Quantification was performed with Quantity One software (Biorad) and the p-value was calculated using Excel (Microsoft). GAPDH was used to determine the equal loading of proteins for all the samples. Click here for file
