# Supplementary material for: Altered proteolytic events in experimental autoimmune encephalomyelitis discovered by iTRAQ shotgun proteomics analysis of spinal cord
Source: Proteome Sci. 2009 Jul 16;7:25. doi: 10.1186/1477-5956-7-25 (PMC2716311; doi:10.1186/1477-5956-7-25)

Peptide sequence: **LWLDSGSEPELR**
Precursor Mass: 1545.743
Protein: **α_1B-glycoprotein**

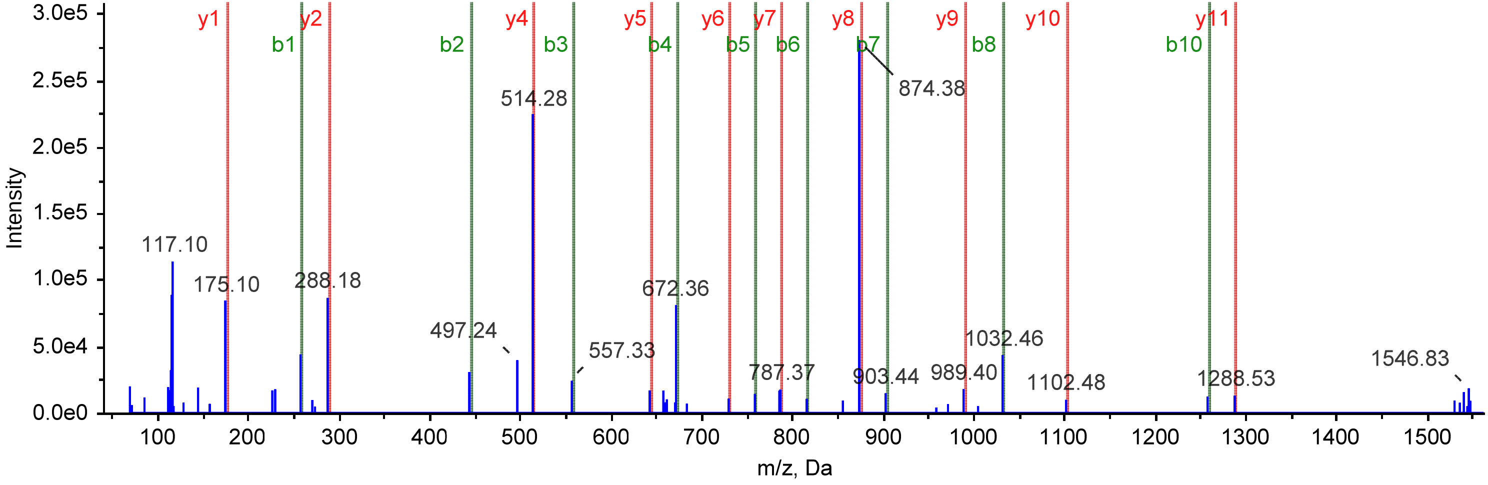

Peptide sequence: **FLELTLPYSVVR**
Precursor Mass: 1580.917
Protein: α_1-**Macroglobulin**

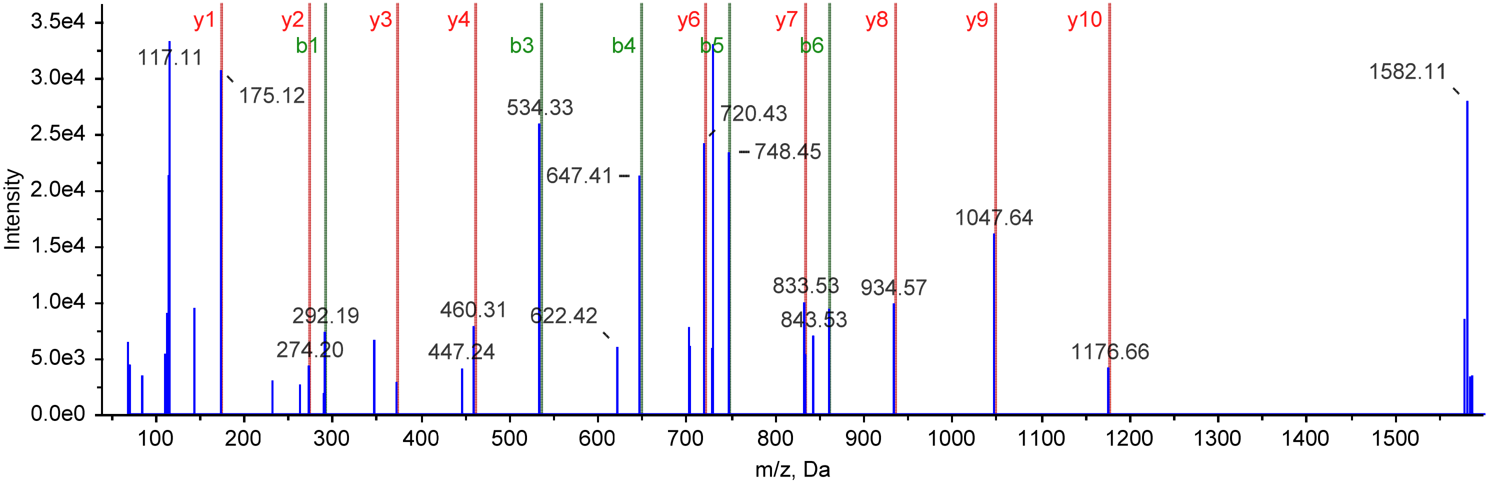

Peptide sequence: **ILAHTEFTPTETDVYACR**

Precursor Mass: 2257.067

Protein: **β_2-Microglobulin**

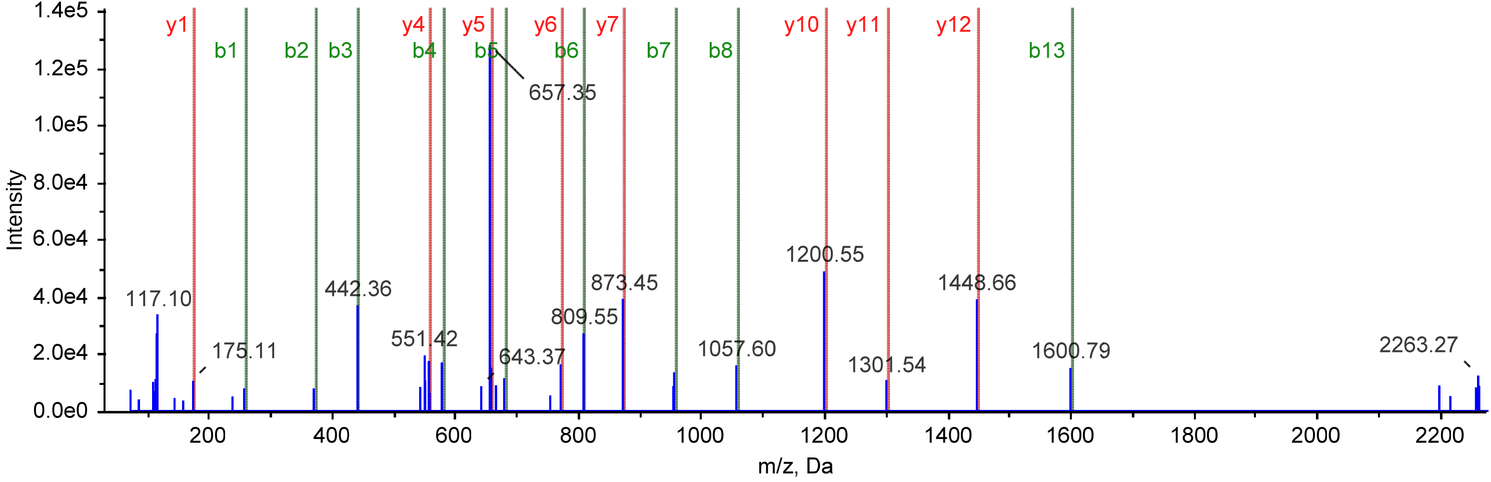

Peptide sequence: **YTSHVQEEQSEVEETIEATK**
Precursor Mass: 2625.292
Protein: **Neurofilament light polypeptide**

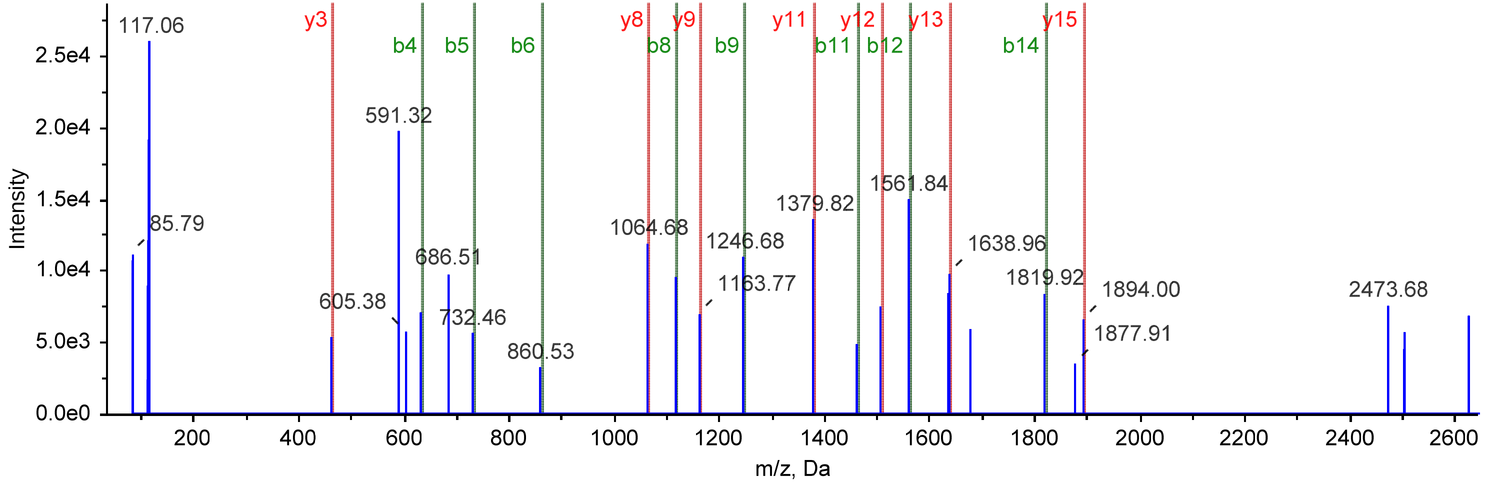

Peptide sequence: **TVEIVDSVEAYATMLR**
Precursor Mass: 1941.006
Protein: **Phosphoglucomutase-1**

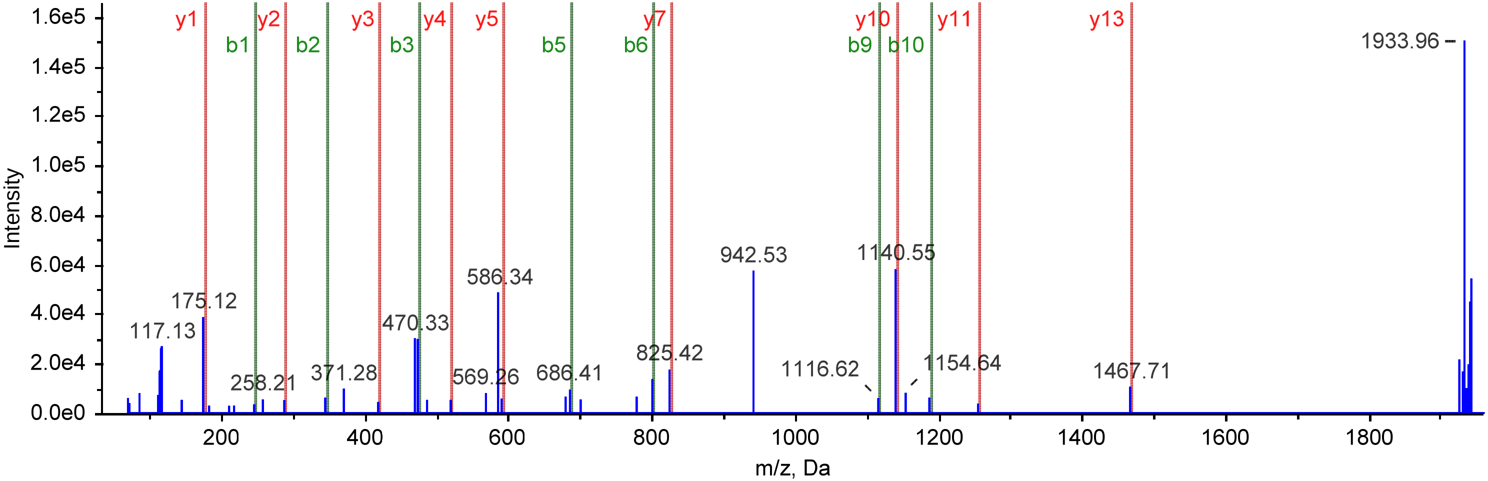

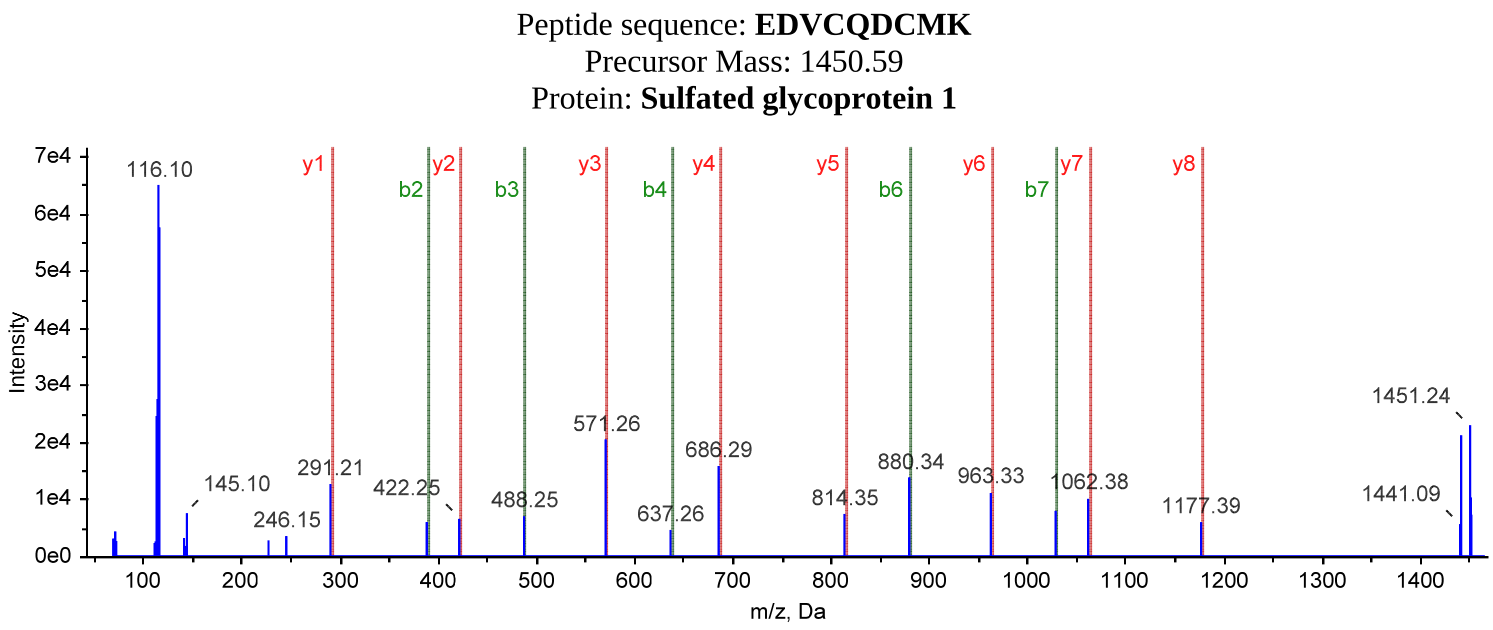
Peptide sequence: **EDVCQDCMK**
Precursor Mass: 1450.59
Protein: **Sulfated glycoprotein 1**
Intensity
0e0
1e4
2e4
3e4
4e4
5e4
6e4
7e4
m/z, Da
100
200
300
400
500
600
700
800
900
1000
1100
1200
1300
1400
y1
y2
y3
y4
y5
y6
y7
y8
b2
b3
b4
b6
b7
116.10
145.10
246.15
291.21
422.25
488.25
571.26
637.26
686.29
814.35
880.34
963.33
1062.38
1177.39
1441.09
1451.24

Peptide sequence: **GFCEVCK**
Precursor Mass: 1165.489
Protein: **Sulfated glycoprotein 1**

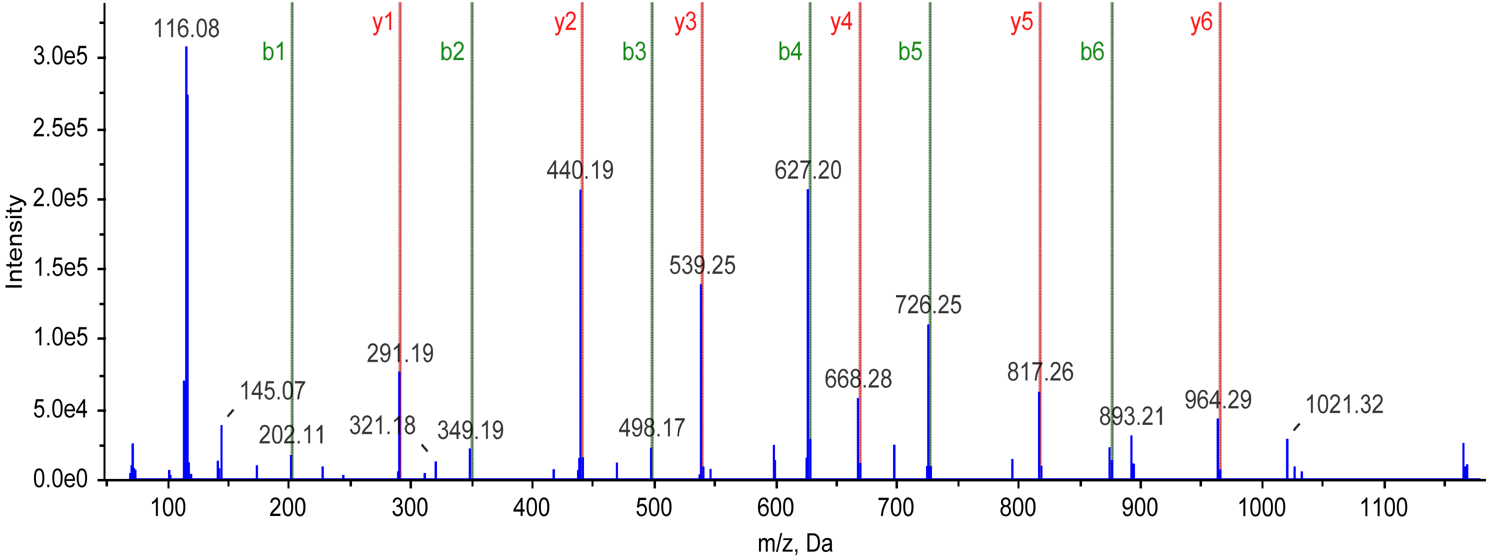

Supplement: Additional file 3 — MS/MS spectra of the semi-tryptic peptides listed in Table 1. All MS/MS spectra were acquired on a 4700 MALDI TOF/TOF tandem MS instrument (ABI). The spectra were matched to proteins by ProteinPilot software (ABI). [file 1477-5956-7-25-S3.pdf]
