# Supplementary material for: Altered proteolytic events in experimental autoimmune encephalomyelitis discovered by iTRAQ shotgun proteomics analysis of spinal cord
Source: Proteome Sci. 2009 Jul 16;7:25. doi: 10.1186/1477-5956-7-25 (PMC2716311; doi:10.1186/1477-5956-7-25)
Supplement: Additional file 4 — Amino acid sequence alignment of the α1 and α2 macroglobulins. The alignment was performed using ClustalW. Rat α1 and α2 macroglobulins showing ~56% of sequence homology. [file 1477-5956-7-25-S4.pdf]

```
sp|Q63041|A1M_RAT  MRRNQLP----IPVFLLLLLLLPRDATAATGKPRYVVLVPSELYAGVPEKVCVHLNHLNE 56
sp|P06238|A2MG_RAT MGKHRLRSLALLPLLLRLLLLLLPTDASAPQKPIYMVMVPSLLHAGTPEKACFLFSHLNE 60
                   * :::*     :*::* *****     ::*. ** *:*:*** *:**.***.*. :.****

sp|Q63041|A1M_RAT  TVTLNVTLEYGVQYSNLLIDQAVDKDSSYCSSFTISRPLS-PSALIAVEIKGPTHHFIKK 115
sp|P06238|A2MG_RAT TVAVRVSLESVRGNQSLFTDLVVDKDLFHCTSFTVPQSSSDEVMFFTVQVKGATHEFRRR 120
                   **::.*:**      ..*: * .****  :*:***:.:. *    :::*::**.**.* ::

sp|Q63041|A1M_RAT  KSMWITKAESPVFVQTDKPIYKPGQTVKFRVVSVDISFRPVNETFPVVYIENPKRNRIFQ 175
sp|P06238|A2MG_RAT STVLVKKKESLVFAQTDKPIYKPGQTVRFRVVSLDESFHPLNELIPLLYIQDPKNNRIAQ 180
                   .:: :.* ** **.*************:*****:* **:*:** :*::**::**.*** *

sp|Q63041|A1M_RAT  WQNVDLPGGLHQLSFPLSVEPALGIYKVVVQKDSGKKIEHSFEVKEYVLPKFEVQVKMPK 235
sp|P06238|A2MG_RAT WQNFNLEGGLKQLSFPLSSEPTQGSYKVVIRTESGRTVEHPFSVEEFVLPKFEVRVTVPE 240
                   ***.:* ***:******* **: * ****::.:**:.:**.*.*:*:*******:*.:*:

sp|Q63041|A1M_RAT  TMAFLEEELVVTACGLYTYGKPVPGLVTMKVCRKYTQSYSNCHGQHSKSICEEFSKQADE 295
sp|P06238|A2MG_RAT TITILEEEMNVSVCGIYTYGKPVPGRVTVNICRKYSNP-SNCFGEESVAFCEKLSQQLDG 299
                   *:::****: *:.**:********* **::****::. ***.*:.* ::**::*:* *

sp|Q63041|A1M_RAT  KGCFRQVVKTKVFQPRQKGYDMKIEVEAKIKEDGTGIELTGTGSCEIANTLSKLKFTKAN 355
sp|P06238|A2MG_RAT RGCFSQLVKTKSFQLKRQEYEMQLDVHAKIQEEGTGVEETGKGLTKITRTITKLSFVNVD 359
                   :*** *:**** ** ::: *:*:::*.***:*:***:* **.*  :*:.*::**.*.:.:

sp|Q63041|A1M_RAT  TFYRPGLPFFGQVLLVDEKGQPIPNKNLTVQVNSVRSQFTFTTDEHGLANILIDTTNFTF 415
sp|P06238|A2MG_RAT SHFRQGIPFVGQVLLVDGRGTPIPYETIFIGADEANLYINTTTDKHGLARFSINTDDIMG 419
                   :.:* *:**.******* :* *** :.: : .:...  :. ***:****.: *:* ::

sp|Q63041|A1M_RAT  SFMGIRVIYKQNNICFDNWWVDEYHTQADHSAARIFSPSRSYIQLELVLGTLACGQTQEI 475
sp|P06238|A2MG_RAT TSLTVRAKYKDSNACYGFRWLTEENVEAWHTAYAVFSPSRSFLHLESLPDKLRCDQTLEV 479
                   : : :*. **:.* *:.  *: * :.:* *:*  :******:::** : ..* *.** *:

sp|Q63041|A1M_RAT  RIHFLLNEDALKDAKDLTFYYLIKARGSIFNSGSHVLPLEQGKVKGVVSFPIRVEPGMAP 535
sp|P06238|A2MG_RAT QAHYILNGEAMQELKELVFYYLMMAKGGIVRAGTHVLPLKQGQMRGHFSILISMETDLAP 539
                   : *::** :*::: *:*.****: *:*.*..:*:*****:**::* .*: * :*..:**

sp|Q63041|A1M_RAT  VAKLIVYTILPNEELIADVQKFDIEKCFANTVNLSFPSAQSLPASDTHLTVKATPLSLCA 595
sp|P06238|A2MG_RAT VARLVLYAILPNGEVVGDTAKYEIENCLANKVDLVFRPNSGLPATRALLSVMASPQSLCG 599
                   **:*::*:**** *::.*. *::**:**.*:* * . ..***: : *:* *:* ***.

sp|Q63041|A1M_RAT  LTAVDQSVLLLKPEAKLSPQSIYNLLPQKAEQGAYLGPLPYKGGEN-CIKAEDITHNGIV 654
sp|P06238|A2MG_RAT LRAVDQSVLLMKPETELSASLIYDLLPVKDLTGFPQGADQREEDTNGCVKQNDTYINGIL 659
                   * ********:***::**.. **:*** *   *   *.    : . * *:* :*   ***:

sp|Q63041|A1M_RAT  YTPKQDLNDNDAYSVFQSIGLKIFTNTRVHKPRYCPMYQAYPPLPYVGEPQALAMSAIPG 714
sp|P06238|A2MG_RAT YSPVQNTNEEDMYGFLKDMGLKVFTNSNIRKPKVCERLRDN--------------KGIPA 705
                   *:* *: *::* *..::.:***:***:.::**: *   :              ..**.

sp|Q63041|A1M_RAT  AGYRSSNIRTSSMMMMGASEVAQEVEVRETVRKYFPETWIWDMVPLDLSGDGELPVKVPD 774
sp|P06238|A2MG_RAT AYHLVSQSHMDAFLESSESPT-------ETRRSYFPETWIWDLVVVDSAGVAEVEVTVPD 758
                   * :  *: : .:::  . * .       ** *.*********:* :* :* .*: *.***

sp|Q63041|A1M_RAT  TITEWKASAFCLSGTTGLGLSSTISHKVFQPFFLELTLPYSVVRGEAFILKATVLNYMPH 834
sp|P06238|A2MG_RAT TITEWKAGAFCLSNDTGLGLSPVVQFQAFQPFFVELTMPYSVIRGEAFTLKATVLNYLPT 818
                   *******.*****. ******..:..:.*****:***:****:***** ********:*

sp|Q63041|A1M_RAT  CIRIHVSLEMSPDFLAVPVGSHEDSHCICGNERKTVSWAVTPKSLGEVNFTATAEALQSP 894
sp|P06238|A2MG_RAT CIRVAVQLEASPDFLAAPEEKEQRSHCICMNQRHTASWAVIPKSLGNVNFTVSAEALNSK 878
                   ***: *.** ******.*   ..: ***** *:*:*.**** *****:****.:****:*

sp|Q63041|A1M_RAT  ELCGNKVAEVPALVQKDTVVKPVIVEPEGIEKEQTYNTLLCPQDAELQENWTLDLPANVV 954
sp|P06238|A2MG_RAT ELCGNEVPVVPEQGKKDTIIKSLLVEPEGLENEVTFNSLLCPMGAEVSELIALKLPSDVV 938
                   *****:*. **    :***::*.::*****:*:* *:*:**** .**:.*  :*.**::**

sp|Q63041|A1M_RAT  EGSARATQSVLGDILGSAMQNLQNLLQMPYGCGEQNMVLFVPNIYVLEYLNETQQLTEAI 1014
sp|P06238|A2MG_RAT EESARASVTVLGDILGSAMQNTQDLLKMPYGCGEQNMVLFAPNIYVLDYLNETQQLTQEI 998
                   * ****: :************ *:**:*************.******:*********: *

sp|Q63041|A1M_RAT  KSKAISYLISGYQRQLNYQHSDGSYSTFGDRGMRHSQGNTWLTAFVLKAFAQAQSYIYIE 1074
sp|P06238|A2MG_RAT KTKAIAYLNTGYQRQLNYKHRDGSYSTFGDKPGRN-HANTWLTAFVLKSFAQARKYIFID 1057
                   *:***:** :********:* *********:   *: :.**********:****:.**:*:

sp|Q63041|A1M_RAT  KTHITNAFNWLSMKQRENGCFQQSGSLLNNAMKGGVDDEVTLSAYITIALLEMPLPVTHS 1134
sp|P06238|A2MG_RAT EVHITQALLWLSQQQKDNGCFRSSGSLLNNAMKGGVEDEVTLSAYITIALLEMSLPVTHP 1117
                   :.***:*: *** :*::****:.**************:****************.*****.

sp|Q63041|A1M_RAT  VVRNALFCLETAWASISN-SQESHVYTKALLAYAFALAGNRAKRSEVLESLNKDAVNEEE 1193
sp|P06238|A2MG_RAT VVRNALFCLDTAWKSARGGAGGSHVYTKALLAYAFALAGNQDTKKEILKSLDEEAVKEED 1177
                   *********:*** *   . :  ******************: .:.*:*:**:::**:**:

sp|Q63041|A1M_RAT  SVHWQRPKNVEENVREMRSFSYKPRAPSAEVEMTAYVLLAYLTSASSRPTRDLSSSDLTT 1253
sp|P06238|A2MG_RAT SVHWTRPQKPSVSV----ALWYQPQAPSAEVEMTAYVLLAYLTTEPAP-----TQEDLTA 1228
                   **** **::  . .*     :: *:*:******************: .:       :..***:

sp|Q63041|A1M_RAT  ASKIVKWISKQQNSHGGFSSTQDTVVALQALSKYGAATFTKSNKEVSVTIESSGTVSGTL 1313
sp|P06238|A2MG_RAT AMLIVKWLTKQQNSHGGFSSTQDTVVALHALSKYGSATFTRAKKAAQVTIHSSGTFSTKF 1288
                   *  ****::*******************:******:****:::*  ..***.****.*  .:

sp|Q63041|A1M_RAT  HVNNGNRLLLQEVRLADLPGNYITKVSGSGCVYLQTSLKYNILPEAEGEAPFTLKVNTLP 1373
sp|P06238|A2MG_RAT QVNNNNQLLLQRVTLPTVPGDYTVKVTGEGCVYLQTSLKYSVLPREE-EFPFTVVVQTLP 1347
                   :***.*:****.* *. :**:* .**:*.***********.:**. * * ***: *:***

sp|Q63041|A1M_RAT  LNFDKAEHHRKFQIHINVSYIGERPNSNMVIVDVKMVSGFIPVKPSVKKLQDQSNIQRTE 1433
sp|P06238|A2MG_RAT GTCEDPKAHTSFQISLNISYTGSRSESNMAIADVKMVSGFIPLKPTVKMLERSVHVSRTE 1407
                    . :..: * .*** :*:** *.*.:***.*.**********:**:** *:  . ::.***

sp|Q63041|A1M_RAT  VNTNHVLIYIEKLTNQTMGFSFAVEQDIPVKNLKPAPVKVYDYYETDEFAIEEYSAPFSS 1493
sp|P06238|A2MG_RAT VSNNHVLIYLDKVSNQTVNLSFTVQQDIPIRDLKPAVVKVYDYYEKDEFAVAKYSAPCST 1467
                   *..******::*::***:.:**:*:****::.**** ********.****:: :**** *:

sp|Q63041|A1M_RAT  DSEQGNA 1500
sp|P06238|A2MG_RAT D--YGNA 1472
                   *   ***
```
