# Supplementary figures and images for: Altered proteolytic events in experimental autoimmune encephalomyelitis discovered by iTRAQ shotgun proteomics analysis of spinal cord
Source: Proteome Sci. 2009 Jul 16;7:25. doi: 10.1186/1477-5956-7-25 (PMC2716311; doi:10.1186/1477-5956-7-25)

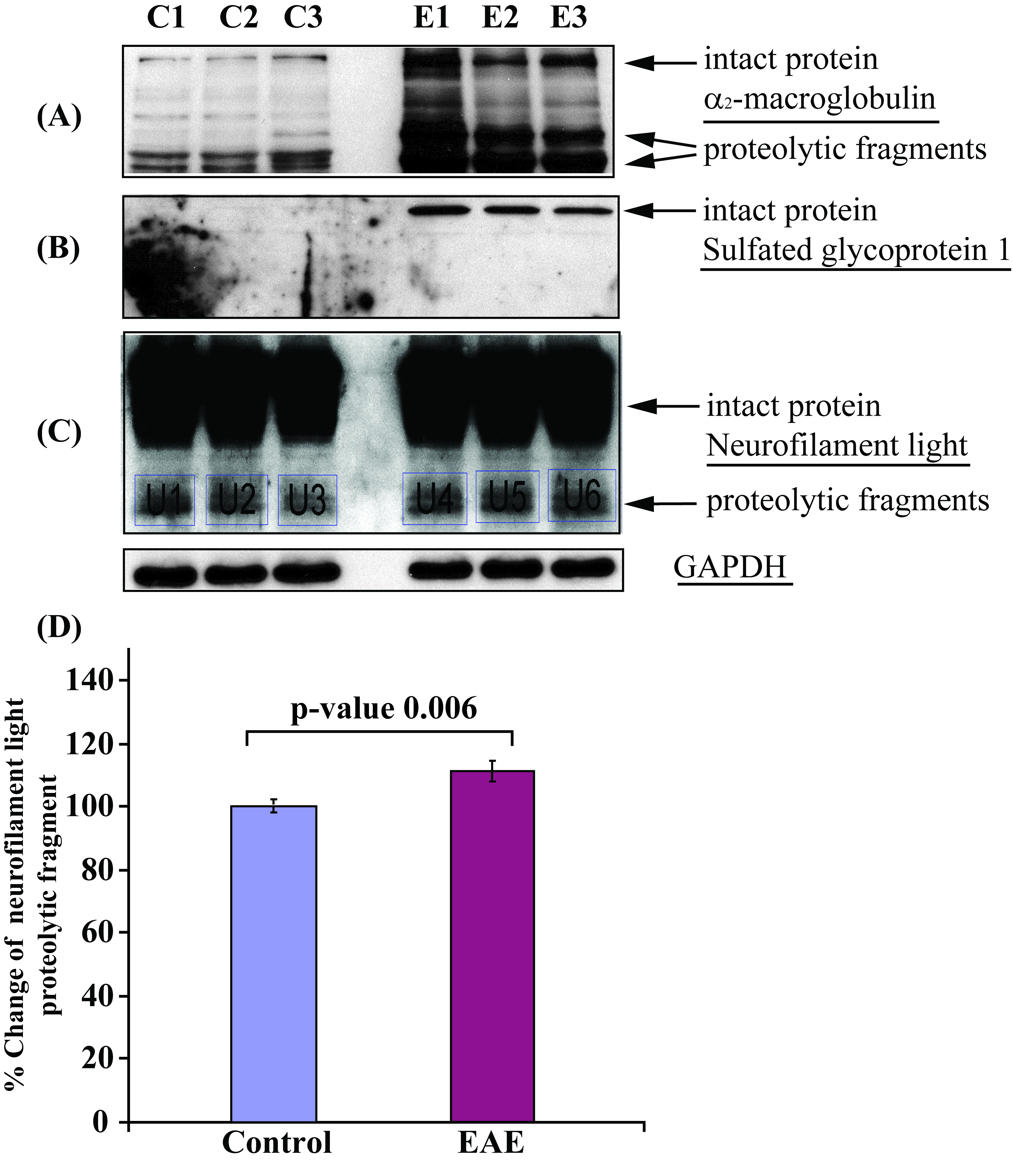

Supplement: Additional file 5 — Western blotting analyses. (A) Longer exposure image of α2-macroglobulin as shown in Fig. 2A. Low levels of the α2-macroglobulin fragments were present in the control spinal cords. More fragments can be seen in EAE samples (B) Sulfated glycoprotein 1 from both control (C1, C2, C3) and EAE (E1, E2, E3) animals. (C) Neurofilament light polypeptide Western blotting and (D) densitometry quantification of neurofilament light protein fragments signals. Quantification was performed with Quantity One software (Biorad) and the p-value was calculated using Excel (Microsoft). GAPDH was used to determine the equal loading of proteins for all the samples. [file 1477-5956-7-25-S5.tiff]
